# Delivery of VEGFA in bone marrow stromal cells seeded in copolymer scaffold enhances angiogenesis, but is inadequate for osteogenesis as compared with the dual delivery of VEGFA and BMP2 in a subcutaneous mouse model

**DOI:** 10.1186/s13287-018-0778-4

**Published:** 2018-01-31

**Authors:** Sunita Sharma, Dipak Sapkota, Ying Xue, Saroj Rajthala, Mohammed A. Yassin, Anna Finne-Wistrand, Kamal Mustafa

**Affiliations:** 1 Department of Clinical Dentistry, Centre for Clinical Dental Research, University of Bergen, 5020 Bergen, Norway; 2 Department of Oral Biology, Faculty of Dentistry, University of Oslo, 0316 Oslo, Norway; 3 The Gade Laboratory for Pathology, Department of Clinical Medicine, University of Bergen, Bergen, Norway; 4 Department of Fibre and Polymer Technology, KTH Royal Institute of Technology, 10044 Stockholm, Sweden

**Keywords:** Bone regeneration, Scaffold, Mesenchymal stem cell, BMP2 and VEGFA, Angiogenesis, Gene delivery

## Abstract

**Background:**

In bone tissue engineering (BTE), extensive research into vascular endothelial growth factor A (VEGFA)-mediated angiogenesis has yielded inconsistent results. The aim of this study was to investigate the influence on angio- and osteogenesis of adenoviral-mediated delivery of VEGFA alone or in combination with bone morphogenetic protein 2 (BMP2) in bone marrow stromal cells (BMSC) seeded onto a recently developed poly(LLA-co-CL) scaffold.

**Methods:**

Human BMSC were engineered to express VEGFA alone or in combination with BMP2 and seeded onto poly(LLA-co-CL) scaffolds. Changes in angiogenic and osteogenic gene and protein levels were examined by quantitative reverse-transcription polymerase chain reaction (RT-PCR), PCR array, and alkaline phosphatase assay. An in vivo subcutaneous mouse model was used to investigate the effect on angio- and osteogenesis of VEGFA alone or in combination with BMP2, using microcomputed tomography (μCT), histology, immunohistochemistry, and immunofluorescence.

**Results:**

Combined delivery of a lower ratio (1:3) of VEGFA and BMP2 (ad-BMP2 + VEGFA) led to upregulation of osteogenic and angiogenic genes in vitro at 3 and 14 days, compared with mono-delivery of VEGFA (ad-VEGFA) and other controls. In vivo, in a subcutaneous mouse model, both ad-VEGFA and ad-BMP2 + VEGFA scaffold explants exhibited increased angiogenesis at 2 weeks. Enhanced angiogenesis was largely related to the recruitment and differentiation of mouse progenitor cells to the endothelial lineage and, to a lesser extent, to endothelial differentiation of the implanted BMSC. μCT and histological analyses revealed enhanced de novo bone formation only in the ad-BMP2 + VEGFA group, corresponding at the molecular level to the upregulation of genes related to osteogenesis, such as *ALPL*, *RUNX2*, and *SPP1*.

**Conclusions:**

Although BMSC expressing VEGFA alone or in combination with BMP2 significantly induced angiogenesis, VEGFA alone failed to demonstrate osteogenic activity both in vitro and in vivo. These results not only call into question the use of VEGFA alone in bone regeneration, but also highlight the importance in BTE of appropriately formulated combined delivery of VEGFA and BMP2.

**Electronic supplementary material:**

The online version of this article (10.1186/s13287-018-0778-4) contains supplementary material, which is available to authorized users.

## Background

Bone tissue engineering (BTE) involves an interplay among mesenchymal stem cells, a supportive scaffold, and the controlled application of growth factors to produce vital bone grafts [1]. Selection of appropriate osteoinductive and/or angiogenic growth factors, a suitable delivery method, and a proper supportive scaffold are critical for a successful outcome in BTE. In addition, the survival and integration of tissue-engineered constructs to the host is critical for the success of BTE and this is largely dependent on adequate vascularization [2]. Therefore, early induction of vascularization is crucial, particularly in large bone defects where the vitality of the implanted cells depends on the vascularity in the scaffolds. The blood vessels surrounding the scaffold-cell construct can provide nutrients by diffusion for distances of 100–200 μm [3]. In this context, various approaches to improve the vascularization of tissue-engineered constructs have been explored [4]. The key events during the early stage of bone repair and regeneration (secretion of angiogenic factors, recruitment of endothelial cells, and stimulation of vascular network formation) are tightly regulated by a complex interplay of several growth factors and molecular regulators [5]. An important research challenge is to harness these crucial events by therapeutic application of angiogenic molecules, such as vascular endothelial growth factor A (VEGFA) [6].

There is some evidence that the current mode of delivery of recombinant growth factors to the defect site might not be a feasible therapeutic option, given the disadvantages of rapid degradation and a short half-life of the growth factors, unpredictable adverse effects, and high cost [7, 8]. In vivo delivery of VEGFA protein for a shorter duration was reported to be less effective in bone repair, and it has been suggested that sustained release of VEGFA during the initial angiogenic phase is necessary for optimal healing of the bone defects [9]. A gene delivery approach is a potential alternative to the protein delivery method to achieve therapeutic concentration of single or multiple growth factors [10]. Gene delivery also allows sustained and regulated delivery of proteins with authentic post-translational modifications at the defect site [11].

Multiple growth factors, including VEGFA and the bone morphogenetic proteins (BMPs; BMP2 to BMP8) have been shown to enhance bone regeneration in a number of model systems [9, 12, 13]. The role of VEGFA in bone repair is attributed to its ability to enhance not only angiogenesis but also osteoblastic activity [9, 14, 15]. The osteoinductive function of BMP2 has been coupled to its ability to induce VEGFA-mediated angiogenesis [16, 17]. As both VEGFA and BMP2 are involved in angiogenesis and osteoinduction and mutually regulate each other’s biological activity, it has been suggested that combined delivery of these growth factors might lead to better outcomes for bone regeneration than mono-delivery of these factors [14, 18].

Accordingly, there are many studies investigating the influence of combined delivery of VEGFA and BMP2 on bone regeneration. However, the reported outcomes are quite inconsistent [16, 19, 20]. For example, using a critical-sized calvarial bone defect model in the mouse, Peng et al. demonstrated that combined delivery of a low ratio (1:5) of VEGFA to BMP2 was more efficient in bone regeneration than individual delivery of either BMP2 or VEGFA [16]. Moreover, although at an early time point (4 weeks) dual delivery resulted in significantly enhanced bone formation compared with individual delivery of either BMP2 or VEGFA, at a later time point (12 weeks) there was no significant difference in bone formation between dual delivery and delivery of BMP2 alone [20]. In contrast, in a rat model, VEGFA was reported to inhibit BMP2 expression and bone formation [21]. These inconsistent outcomes clearly support the need for further studies to optimize the amount and mode of delivery of VEGFA and BMP2 in bone regeneration.

Another important component of BTE, the scaffold, acts not only as a carrier for cells and growth factors, but also functions as a supporting matrix on which cells can differentiate and form mineralized tissue. Ideally, scaffolds should remain at the defect site, gradually degrading as the new bone forms [22]. Degradable poly(L-lactide-co-є-caprolactone) (poly(LLA-co-CL)) scaffold, developed by our research group, has been extensively evaluated for its applicability in BTE [23–25]. Using adenoviral-mediated delivery of BMP2 in human bone marrow stromal cells (BMSC) seeded onto poly(LLA-co-CL) scaffolds, we have previously shown that this system is feasible and efficient in angiogenesis and bone regeneration [13]. In the current study, using the previously established model, we aimed to investigate the role of adenoviral-mediated delivery of VEGFA alone and in combination with BMP2 (VEGFA:BMP2 = 1:3) in angiogenesis and in bone formation. The results showed that although BMSC expressing VEGFA alone or in combination with BMP2 significantly induced angiogenesis, VEGFA alone failed to demonstrate osteogenic activity both in vitro and in vivo.

## Methods

### Cell culture

Well-characterized primary human BMSC were purchased from StemCell Technologies (cat. number: MSC-001 F; Vancouver, British Columbia, Canada) and expanded using a MesenCult Proliferation Kit (Stem Cell Technologies, Part ID 05411) following the standard culture protocol. All cell culture experiments were carried out in a humidified atmosphere at 37 °C and 5% CO_2_. For validation of the changes in gene expression induced by adenoviral-mediated delivery of VEGFA alone or in combination with BMP2, commercially available human BMSC from two additional donors were used (henceforth referred to as donor 2 and 3 BMSC), as described in the supporting information (Additional file 1).

### Preparation of BMSC-seeded scaffolds

Poly(LLA-co-CL) scaffolds were fabricated using the solvent-casting-salt-leaching method as described previously [23, 26]. For the in vitro experiments, scaffolds of diameter ≈ 12 mm, height ≈ 1.3 mm, porosity 85%, and pore size 90–500 μm, as determined by microcomputed tomography (μCT), were placed on the bottom of 48-well plates, pre-wetted with the culture medium, and incubated overnight in a humidified atmosphere at 37 °C and 5% CO_2_. BMSC were seeded onto the scaffolds at a density of 5 × 10^4^ cells/scaffold.

### Adenoviral expression vector construction and transduction of BMSC

Replication-deficient adenoviral expression vectors carrying the coding sequences of *VEGF165* (reference sequence: NM_001025370.2) (ad-VEGFA) and *BMP2* gene (reference sequence: NM_001200.2) (ad-BMP2) were purchased from Cyagen Biosciences Inc. In the ad-VEGFA construct, a gene encoding for *DsRed* was used as a marker, and in the ad-BMP2 construct, a gene encoding for enhanced green fluorescent protein (eGFP) was used. An adenoviral vector carrying only the *eGFP* coding sequence (ad-GFP) served as a control. Adenoviral particles were generated by transfecting HEK 293 cells (ATCC-CRL-1573) with Pac I digested constructs. Early passage (passages 2–3) BMSC were infected in monolayer culture with respective adenoviruses (multiplicity of infection (MOI) = 100) to attain 80–90% infection efficiency, confirmed by fluorescent microscopy. BMSC infected with ad-GFP, ad-VEGFA, and a mixture of ad-VEGFA and ad-BMP2 (ratio 1:3) are henceforth referred to as ‘ad-GFP BMSC’, ‘ad-VEGFA BMSC’ and ‘ad-BMP2 + VEGFA BMSC’, respectively. Based on the results from previous studies ([14] and references therein), the current study utilized a lower (1:3) ratio of ad-VEGFA and ad-BMP2 viral particles (25 MOI of ad-VEGFA and 75 MOI of ad-BMP2) for the combined BMSC group. After 48 h of infection in monolayer culture with the respective adenoviral particles, BMSC were seeded at a density of 5 × 10^4^ cells/scaffold. BMSC grown in scaffolds were harvested after 3 and 14 days for mRNA and protein expression analyses. Culture supernatants were also collected at the respective time points for enzyme-linked immunosorbent assay (ELISA).

### Total RNA extraction

Total RNA from the in vitro seeded scaffolds was extracted using Maxwell® 16 LEV simplyRNA Kit (cat no.: AS1270; Promega) on a Maxwell® 16 instrument in accordance with the manufacturer’s protocol. The quantity and purity of the total RNA were determined using a Nanodrop Spectrophotometer (ThermoScientific Nano Drop Technologies, Wilmington, DE, USA). The Agilent 2100 Bio analyzer (Agilent Technologies) was used to examine the integrity of RNA.

### Expression analysis of osteogenesis- and angiogenesis-related genes in vitro using polymerase chain reaction (PCR) array

To examine the range of genes modulated by the expression of VEGFA and the co-expression of VEGFA and BMP2 in BMSC in the three-dimensional scaffold (in vitro), a custom PCR array (cat no.: 330131; SuperArray Bioscience, Frederick, MD, USA) containing primer pairs for 30 genes related to osteogenesis and angiogenesis was used. Total RNA from three biological replicates (*n* = 3) of ad-GFP, ad-VEGFA, and ad-BMP2 + VEGFA groups at 3 and 14 days was used for cDNA synthesis. PCR amplification was performed using the following cycling conditions: 95 °C for 10 min, (95 °C for 15 s, and 60 ° C for 1 min) × 40 cycles in ABI Prism Sequence Detector 7900 HT (Applied Biosystems, Foster City, USA). Pre- and post- PCR quality control measures, as recommended by the manufacturer, were strictly followed. PCR array data were analyzed as described previously [27]. Briefly, threshold cycle (Ct) was used to calculate the 2^–ΔCt^ value for each gene using PCR Array Data Analysis Web Portal (SABiosciences). 2^–ΔCt^ values were then exported to microarray data analysis software (J-Express 2012). For statistical analysis, unsupervised hierarchical clustering and one-way analysis of variance (ANOVA) with Bonferroni post hoc analysis were used.

### Reverse-transcription (RT) and quantitative RT-PCR (qRT-PCR) using TaqMan assays

Three hundred nanograms of total RNA were converted to cDNA by reverse-transcription reaction using a high capacity cDNA Archive Kit (Applied Biosystems, Carlsbad, CA, USA). *VEGFA* (Hs00900055_m1) and *BMP2* (Hs00154192_m1) TaqMan assays were used to verify the expression of *VEGFA* and *BMP2* mRNA levels in adenovirus-transduced BMSC. The in vitro PCR array results were independently validated by qRT-PCR, using TaqMan assays for selected key genes: *ALPL* (Hs01029144_m1), *RUNX2* (Hs00231692_m1), and *SPP1* (Hs00959010_m1). *GAPDH* (Hs99999905_m1) was used as an endogenous control. All qRT-PCR amplifications were performed on an ABI Prism Sequence Detector 7900 HT (Applied Biosystems, Foster City, USA) under standard cycling conditions. A comparative 2^–ΔΔCt^ method was used to quantify the relative mRNA expression.

### Alkaline phosphatase (ALP) staining

ALP staining was performed to analyze the osteoblastic differentiation potential of BMSC transduced with respective adenoviral particles. After 48 h of infection the cells were trypsinized and 2 × 10^4^ cells were seeded in a monolayer on a four-well culture dish. ALP staining was performed at 3 and 14 days. Briefly, cells were washed with phosphate-buffered saline (PBS) and stained for ALP activity using Napthol AS-TR phosphate and fast red violet B salt (Sigma-Aldrich) as described previously [28].

### BMP2 ELISA

Culture supernatants from the transduced BMSC seeded onto scaffolds were collected at 3 and 14 days for ELISA analysis of BMP2 (cat no.: DBP200; R and D systems). ELISA was performed in duplicates following the manufacturer’s instructions.

### In vivo subcutaneous implant model in nonobese diabetic/severe combined immunodeficiency (NOD/SCID) mice

#### Preparation of scaffold implants

Poly(LLA-co-CL) scaffolds (diameter ≈ 6 mm, height ≈ 1.3 mm) were placed at the bottom of 96-well plates, pre-wetted with the culture medium, and incubated overnight in a humidified atmosphere at 37 °C and 5% CO_2_. BMSC (5 × 10^5^) were seeded onto each scaffold after 48 h of infection with the respective adenoviral particles, incubated overnight in a humidified atmosphere at 37 °C and 5% CO_2_, and implanted subcutaneously in NOD/SCID mice. Scaffolds seeded with untransduced BMSC (untransduced group) and scaffolds without BMSC (scaffold-only group) were used as additional controls in the in vivo experiments.

#### Surgical implantation of scaffolds

All animal experiments were approved by the Norwegian Animal Research Authority and conducted according to the European Convention for the Protection of Vertebrates used for Scientific Purposes (local approval number 4940). Twenty NOD-SCID mice (6–8 weeks old; Taconic Farms, Denmark) (10 mice each for the 2- and 8-week time points) were used for subcutaneous implantation of scaffolds. The animals were anesthetized by subcutaneous injection of midazolam 5 mg/ml hyponorm solution. Two midline surgical incisions approximately 2 cm in length were made on the backs of the mice and extended laterally by blunt dissection to create a subcutaneous pouch. Each animal received four randomly allocated scaffold implants from the following groups: i) ad-GFP (9 replicates); ii) ad-VEGFA (9 replicates); iii) ad-VEGF + ad-BMP2 (9 replicates); iv) untransduced (8 replicates); and v) scaffold-only group (5 replicates), as described previously [13]. The wounds were closed with Histoacryl Tissue Adhesive (*n*-butyl cyanoacrylate) (3 M; St. Paul, MN, USA). At 2 and 8 weeks, the animals were euthanized by CO_2_ inhalation and subsequent cervical dislocation. The scaffolds retrieved at the 2-week time point were cut in half and used for: i) formalin fixation (fixed in 4% buffered formalin and decalcified in 12.5% EDTA and 2.5% PFA in PBS) and paraffin embedding; ii) RNA extraction (stored in RNA later, Ambion); and iii) cryosection (embedded in Tissue-Tek). In total, 7 cut halves (replicates) were used for formalin fixation, 6 for RNA extraction, and 5 for cryosection for the ad-GFP, ad-VEGFA, and ad-BMP2 + VEGFA groups. For the untransduced group, 6 replicates each were used for RNA extraction and formalin fixation and 4 for cryosection. For the scaffold-only group, 3 replicates each were used for RNA extraction and cryosection, and 4 for formalin fixation. Of the scaffolds retrieved at 8 weeks, 3 randomly selected scaffolds from all the groups were used for μCT analysis. All remaining scaffolds were formalin fixed and paraffin embedded (FFPE) for histological analysis.

### Expression analysis of osteogenesis- and angiogenesis-related genes in the in vivo scaffold explants

For in vivo scaffold explants (2 weeks), 600 ng total RNA from six biological replicates (*n* = 6) of ad-GFP, ad-VEGFA, and ad-BMP2 + VEGFA BMSC groups were converted to first-strand cDNA using the RT^2^ First Strand Kit (C-03; SABiosciences, Frederick, MD, USA). To examine the range of genes modulated by BMP2 overexpression, a custom PCR array (cat no.: 330131; SuperArray Bioscience, Frederick, MD, USA) containing primer pairs for 30 genes related to osteogenesis and angiogenesis was used. PCR amplification and statistical analyses were performed as described above for the in vitro scaffolds.

### Histological analysis

The FFPE specimens were cut into 5-μm sections. The sections were deparaffinized in xylene, rehydrated in graded ethanol, and stained with hematoxylin and eosin (H&E). The stained sections were dehydrated, cleared, and mounted using EuKit mounting medium.

### CD31 immunohistochemistry (IHC)

To examine the contribution to angiogenesis in the explants of cell-autonomous endothelial differentiation of implanted BMSC, IHC targeting human CD31 protein was performed on 5-μm thick FFPE tissue specimens of in vivo scaffold explants harvested at 2 and 8 weeks. Briefly, antigen retrieval was performed by microwave treatment in citrate buffer, pH 6.0 (S1699, DAKO). After blocking with 10% goat serum, monoclonal mouse anti-human CD31 primary antibody (clone JC70A, DAKO; 1:50 dilution) was applied. After washing, anti-mouse secondary antibody conjugated with horseradish peroxidase-labeled polymer (EnVision System, DAKO) was applied. The presence of antigen was visualized by staining with 3,3’-diaminobenzidine (DAKO), counterstained with hematoxylin (DAKO), and mounted with EuKit mounting medium. Sections incubated with 3% bovine serum albumin (BSA) instead of primary antibody served as negative controls. FFPE tissues from human oral mucosa served as positive controls. The normal human oral mucosa specimen was collected after informed written patient consent and was approved by the Committee for Medical and Health Research Ethics in West Norway (ref 2011/1244/REK vest).

### CD31 immunofluorescence (IF)

IF targeting mouse CD31 protein was performed on 8-μm thick frozen tissue specimens of in vivo scaffold explants harvested at 2 weeks. Briefly, the sections were fixed in cold acetone (50%, –20 °C) for 10 min, blocked with 10% goat serum in 3% BSA in PBS and incubated with rat anti-mouse CD31 (#550274, BD Pharminogen™; 1:250 dilution) for 1 h at room temperature. After washing, goat anti-rat secondary antibody conjugated with Alexa Fluor 546 (cat. no.: A-11081; ThermoFisher Scientific, 1:200 dilution) was applied for 30 min in the dark. The sections were washed and mounted in ProLong® Gold antifade reagent with DAPI (cat no.: P36935; Invitrogen). Specimens were examined by confocal laser microscopy (Leica TCS SP5, Leica Microsystems, Germany). At least three randomly selected areas, including the periphery and interior of the explants, were captured from each specimen. The percentage of CD31-positive areas in the captured image was calculated using Imaris x64 software (version 8.4.0).

### Western blot analysis

Twenty-five micrograms of protein was resolved in NuPAGE® Novex 4–10% Bis-Tris gel (Life Technologies, NY, USA) in NuPAGE® MOPS/MES SDS running buffer (Life Technologies, NY, USA) and immunoblotted with monoclonal mouse anti human-VEGF antibody (sc-7269; Santa Cruz Biotechnology; 1:500 dilution). Anti-GAPDH antibody (sc-25778; Santa Cruz; 1:5000 dilution) served as a loading control. The blots were visualized by enhanced chemiluminescence (Supersignal® West pico; Pierce Biotechnology, Rockford, IL, USA) and images were detected in a Fujifilm Las-4000 scanner.

### Microcomputed tomography analysis

μCT analysis was carried out by two of the authors (YX and MAY). Interobserver agreement was high. The in vivo scaffold explants harvested at 8 weeks were scanned with a Skyscan 1172 x-ray μCT imaging system (Bruker, Kontich, Belgium) at 7-μm resolution. The x-ray source was operated at 50 kV and 200 μA, with a 0.5-mm aluminum filter. Two-dimensional CT images were captured every 0.4° through 360° rotation and then reconstructed by Skyscan NRecon software at thresholds of 0.0—0.125. Regions of interest were selected, and three-dimensional analysis was done using Skyscan Ctan software. Newly formed bone was segmented from the background using a global threshold of 85–255 and the morphometric parameter of bone volume (mm^3^) was measured by Ctan.

### Statistical analyses

Data are expressed as mean ± standard error of the mean (SEM). ANOVA test with Bonferroni post hoc analysis was used for comparison of the means of multiple groups. Statistical analyses were performed using GraphPad Prism software version 5.00 for Windows (GraphPad Software, San Diego, CA, USA; www.graphpad.com), with the level of significance set at 5%.

## Results

### VEGFA and BMP2 adenoviral expression vectors upregulated *VEGFA* and *BMP2* mRNA and respective protein levels

**Fig. 1:**
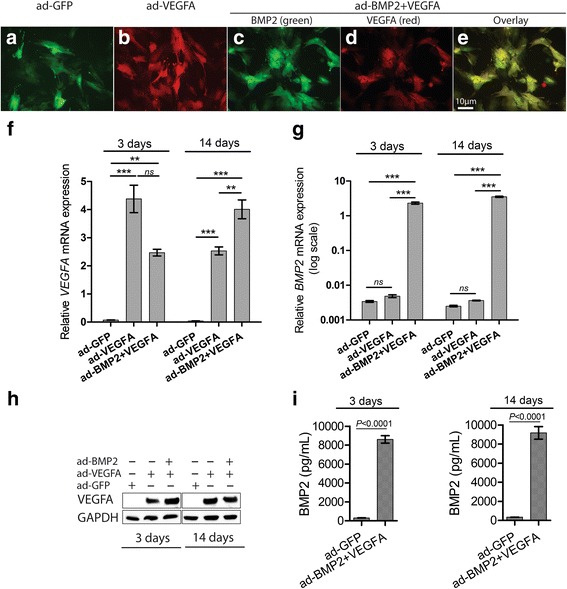
VEGFA and BMP2 adenoviral vectors (ad) respectively upregulated the expression of vascular endothelial growth factor A (VEGFA) and bone morphogenetic protein 2 (BMP2) in BMSC. Representative immunofluorescence images of BMSC in monolayer transduced with ad-GFP (**a**), ad-VEGFA (**b**), and ad-VEGFA + BMP2 (**c**–**e**) adenoviral particles. BMSC (5× 10^4^) were seeded in each scaffold and harvested after 3 and 14 days for mRNA and protein analyses. Significant upregulation of *VEGFA* mRNA (**f**) and VEGFA protein (**h**) levels were found at both time points in both ad-VEGFA and ad-BMP2 + ad-VEGFA BMSC compared with the ad-GFP BMSC. Similarly, significant upregulation of *BMP2* mRNA (**g**) was found in ad-BMP2 + VEGFA BMSC compared with the ad-GFP and ad-VEGFA BMSC at both time points. *VEGFA* and *BMP2* mRNA levels were normalized to *GAPDH* mRNA level. **i** ELISA disclosed higher levels of secreted BMP2 in the culture supernatant of ad-BMP2 BMSC compared with the ad-GFP BMSC at both 3 and 14 days. Error bars in (**f**) and (**g**) represent SEM of three repeated experiments (*n* = 3) performed in three technical replicates. ANOVA test with Bonferroni post hoc analysis was used for statistical analysis in (**f**) and (**g**) and Student’s *t* test was performed in (**i**). Error bars in (**i**) represent SEM of three repeated experiments (*n* = 3). ***p < 0.001, **p < 0.01. GFP green fluorescent protein, ns not significant

Transduction of BMSC with ad-GFP, ad-VEGFA, and ad-BMP2 viral particles was efficient (80–90%) and the transduced cells demonstrated similar morphology in monoculture (Fig. 1a–e). Compared to the control ad-GFP BMSC, ad-VEGFA and ad-BMP2 + VEGFA BMSC seeded onto scaffolds expressed significantly higher levels of *VEGFA* mRNA and protein at day 3 and day 14 (Fig. 1f, h). Similarly, significantly higher levels of *BMP2* mRNA and secreted BMP2 protein were found in ad-VEGFA + BMP2 BMSC seeded onto scaffolds at day 3 and 14, compared with the control ad-GFP and ad-VEGFA BMSC (Fig. 1 g, i).

### Adenoviral-mediated combined delivery of VEGFA and BMP2 was associated with upregulation of osteogenic and angiogenic markers in vitro

**Fig. 2:**
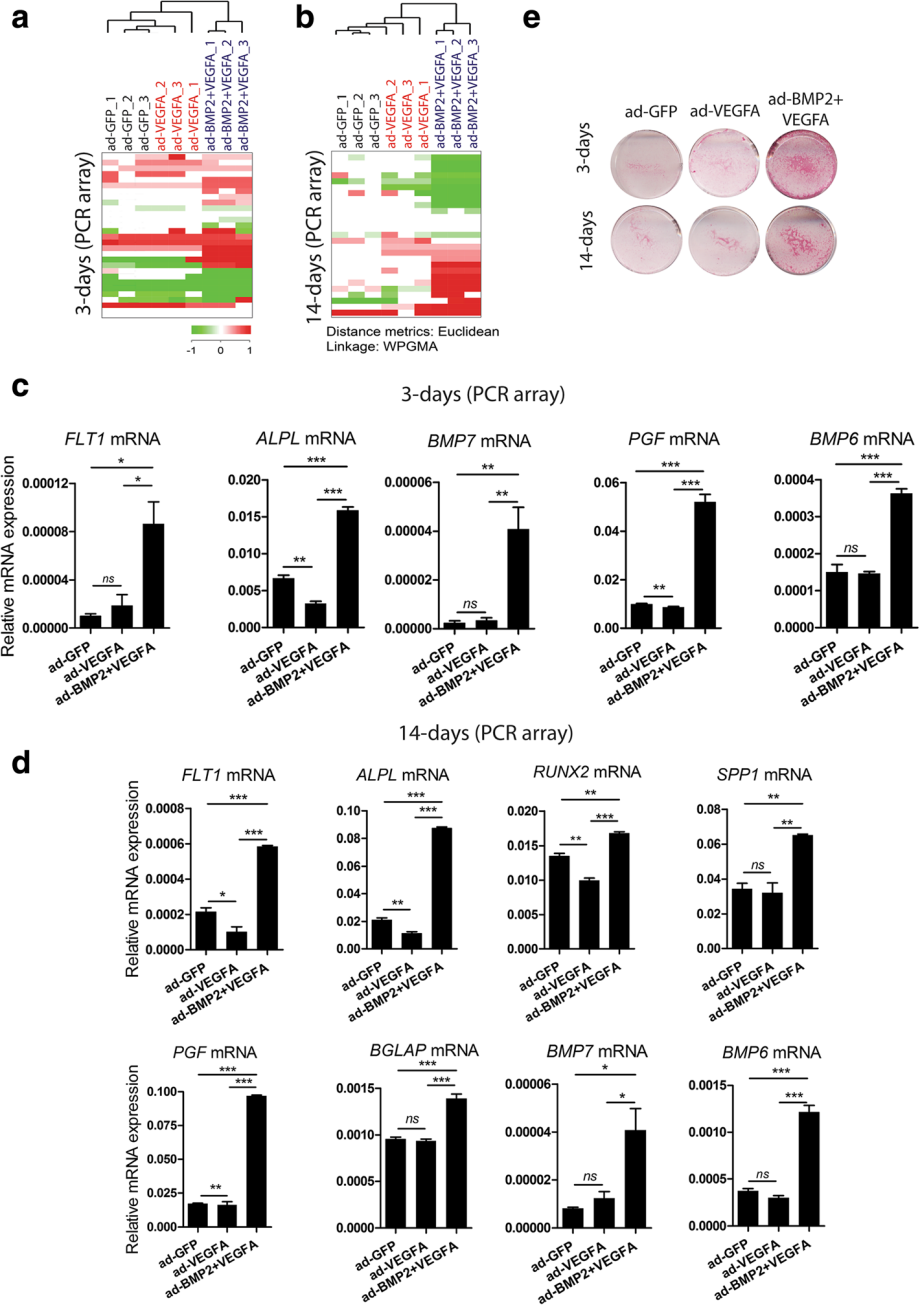
Adenoviral (ad) vector-mediated combined delivery of bone morphogenetic protein 2 (BMP2) and vascular endothelial growth factor A (VEGFA) led to upregulation of osteogenic and angiogenic molecules in vitro*.* ad-GFP, ad-VEGFA, or ad-BMP2 + VEGFA BMSC (5 × 10^4^) were seeded in each scaffold and harvested at 3 and 14 days for custom polymerase chain reaction (PCR) array and TaqMan-based qRT-PCR. **a**, **b** Unsupervised hierarchical clustering demonstrated two separate clusters consisting of replicates of ad-BMP2 + VEGFA, and replicates of ad-GFP and ad-VEGFA BMSC at both time points. **c** ANOVA disclosed that *ALPL*, *BMP7*, *BMP6*, *FLT1*, and *PGF* mRNA levels were found to be significantly upregulated at 3 days in ad-BMP2 + VEGFA BMSC. **d** More osteogenic markers were significantly induced at 14 days in ad-BMP2 + VEGFA BMSC compared with the ad-GFP and ad-VEGFA BMSC groups. Error bars represent SEM of three biological replicates (*n* = 3) performed in three technical replicates. ANOVA test with Bonferroni post hoc analysis was applied in **c** and **d. e** Representative images demonstrating higher alkaline phosphatase activity in ad-BMP2 + VEGFA BMSC in monolayer culture as compared with the ad-GFP and ad-VEGFA BMSC at 3 and 14 days. Experiments were repeated at least three times. ***p < 0.001, **p < 0.01, *P < 0.05. GFP green fluorescent protein, ns not significant

Custom PCR array was used to examine differentially expressed osteogenesis- and angiogenesis-related genes with adenoviral-mediated delivery of VEGFA alone or in combination with BMP2. Unsupervised cluster analysis showed that replicates of ad-BMP2 + VEGFA BMSC grown on scaffolds clustered separately from those of ad-GFP and ad-VEGFA BMSC on both days 3 and 14 (Fig. 2a, b). Correspondingly, the ANOVA test with Bonferroni post hoc analysis showed significant upregulation of the osteogenic markers *ALPL*, *BMP7*, and *BMP6* at day 3, and *ALPL*, *RUNX2*, *SPP1*, *BGLAP*, *BMP6*, and *BMP7* at day 14 in ad-BMP2 + VEGFA BMSC compared with the ad-GFP and ad-VEGFA BMSC (Fig. 2c, d). Additionally, mRNA expression levels of angiogenesis-related molecules such as *FLT1* (VEGFR1) and its ligand *PGF* were significantly upregulated only in ad-BMP2 + VEGFA BMSC as compared with the ad-GFP and ad-VEGFA BMSC (Fig. 2c, d). It is of interest to note that none of the angiogenesis-related markers examined were significantly overexpressed in ad-VEGFA BMSC. The mRNA expression level of *PECAM1* (encoding CD31 protein) did not differ significantly among the different groups at either 3 or 14 days (data not shown). To confirm the induction of *ALPL* mRNA at the protein level, ALP staining was performed. The ALP level was found to be highly induced at day 3 and persisted at day 14 in ad-BMP2 + VEGFA BMSC, in contrast to the comparatively low ALP activity in ad-GFP and ad-VEGFA BMSC (Fig. 2e). Independent validation of the differentially expressed selected genes, as identified by PCR array, was performed by qRT-PCR using TaqMan assays for *ALPL*, *RUNX2*, and *SPP1*. Consistent with the PCR array results, *ALPL* was found to be significantly upregulated in ad-BMP2 + VEGFA BMSC at day 3 (Additional file 2: Figure S1A). Similarly, at day 14 mRNA levels of *ALPL*, *RUNX2*, and *SPP1* were significantly higher in ad-BMP2 + VEGFA compared with those of ad-GFP and ad-VEGFA BMSC (Additional file 2: Figure S1B–D). Furthermore, overexpression of mRNA levels of *ALPL* and *RUNX2* associated with the adenoviral-mediated combined delivery of BMP2 and VEGFA was confirmed in BMSC from two further donors (donor 2 and donor 3), as described in Additional file 1 and Additional file 2 (Figure S1E–R).

### Adenoviral-mediated delivery of VEGFA alone or in combination with BMP2 was associated with enhanced angiogenesis in scaffold explants in NOD/SCID mice

**Fig. 3:**
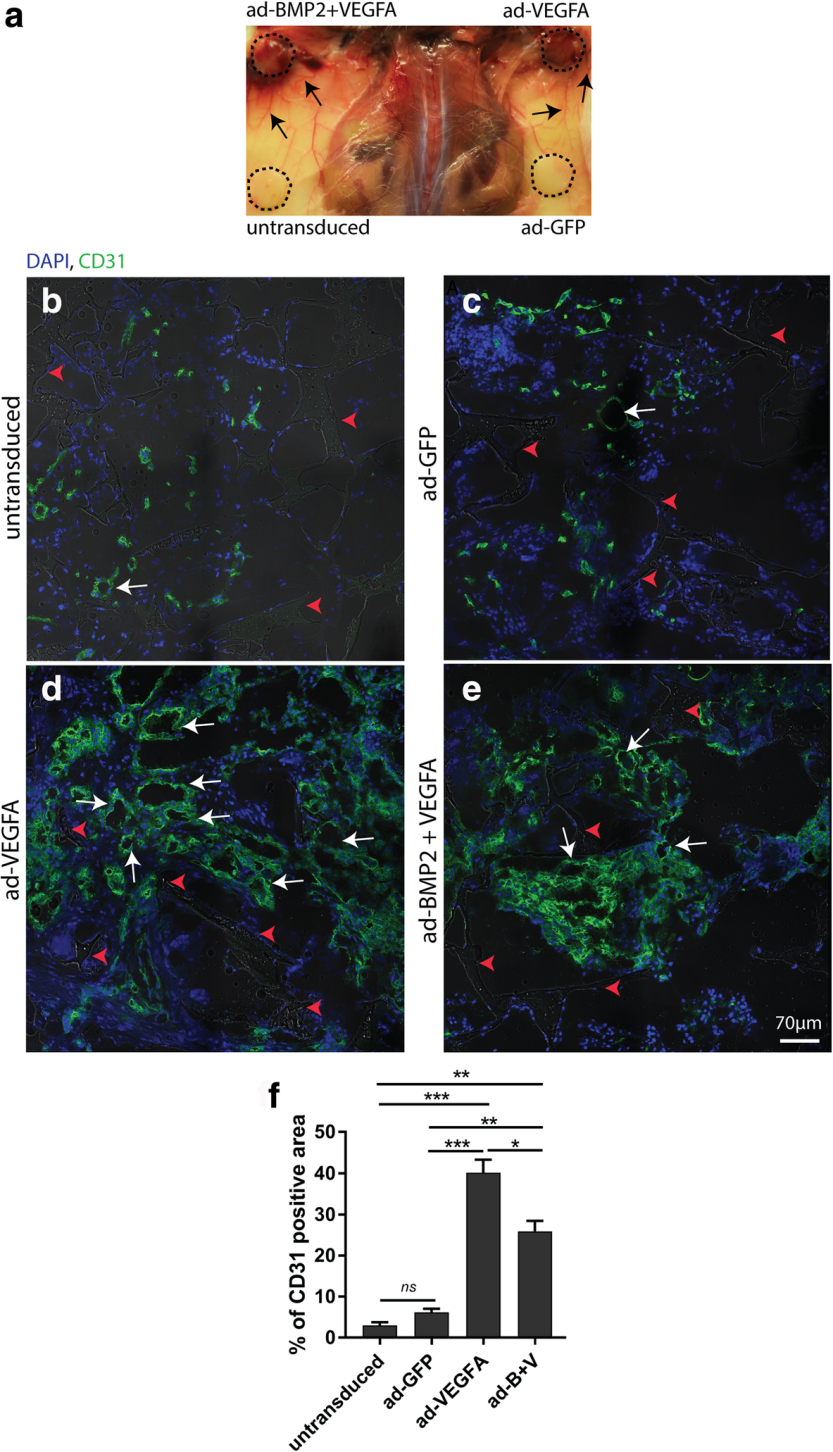
Adenoviral (ad) vector-mediated expression of vascular endothelial growth factor A (VEGFA) alone and in combination with bone morphogenetic protein 2 (BMP2) was associated with enhanced angiogenesis in scaffold explants. **a** Representative images at 2 weeks revealed more reddish scaffold explants with multiple capillary/vessel like structures (black arrows) radiating from the periphery in ad-VEGFA and ad-BMP2 + VEGFA groups compared with the other control explants. **b**–**e** Frozen sections of scaffold explants at 2 weeks were stained with anti-CD31 targeting mouse CD31 protein and examined for capillary-like structures. Few CD31-positive structures were found in untransduced and ad-GFP scaffold explants (**b** and **c**, white arrows). However, in ad-VEGFA and ad-BMP2 + VEGFA explants, many CD31-positive capillary-like structures (white arrows) were seen both at the periphery (not shown in the figure) as well as within the scaffolds (**d**, **e**). Red arrowheads indicate remnants of the scaffold material. **f** Quantification of CD31-positive area in the scaffold explants at 2 weeks demonstrated significantly more CD31-positive structures in ad-VEGFA and ad-BMP2 + VEGFA (ad-B + V) scaffolds than in the untransduced and ad-GFP explants. ANOVA test with Bonferroni post hoc analysis was used for statistical analysis in (**e**). Error bars represent SEM. ***p < 0.001; **p < 0.01; *P < 0.05. GFP green fluorescent protein, ns not significant

The angiogenic effect of the delivery of VEGFA alone or in combination with BMP2 in scaffold explants was next examined using CD31 IF targeting mouse endothelial cells. Visual inspection disclosed that ad-VEGFA and ad-BMP2 + VEGFA scaffold explants were more reddish in color, with the presence of many capillary/vessel-like structures compared with the control explants at both at 2 and 8 weeks (8-week data not shown) (Fig. 3a). IF targeting mouse CD31 protein revealed more CD31-positive areas with multiple capillary-like structures in ad-VEGFA (40.17 ± 3.17%) and ad-BMP2 + VEGFA (25.89 ± 2.5%) compared with untransduced (2.98 ± 0.8%) and ad-GFP (6.14 ± 088%) explants at 2 weeks (Fig. 3b–f). To examine the contribution to angiogenesis of cell-autonomous endothelial differentiation of implanted BMSC in the explants, IHC targeting human CD31 protein was performed on FFPE scaffold explants retrieved at 2 and 8 weeks. Except for ad-VEGFA BMSC explants at 8 weeks (Additional file 3: Figure S2E), no CD31 positivity was observed in the capillary- or vessel-like structures on the entire scaffold explants for all groups at both 2 and 8 weeks (Additional file 3: Figure S2). Correspondingly, the level of expression of human *PECAM* mRNA was slightly but insignificantly higher in ad-VEGFA explants at 2 weeks (data not shown).

### Adenoviral vector-mediated co-expression of VEGFA and BMP2 induced ectopic bone formation in scaffold explants in subcutaneous NOD/SCID mice model

**Fig. 4:**
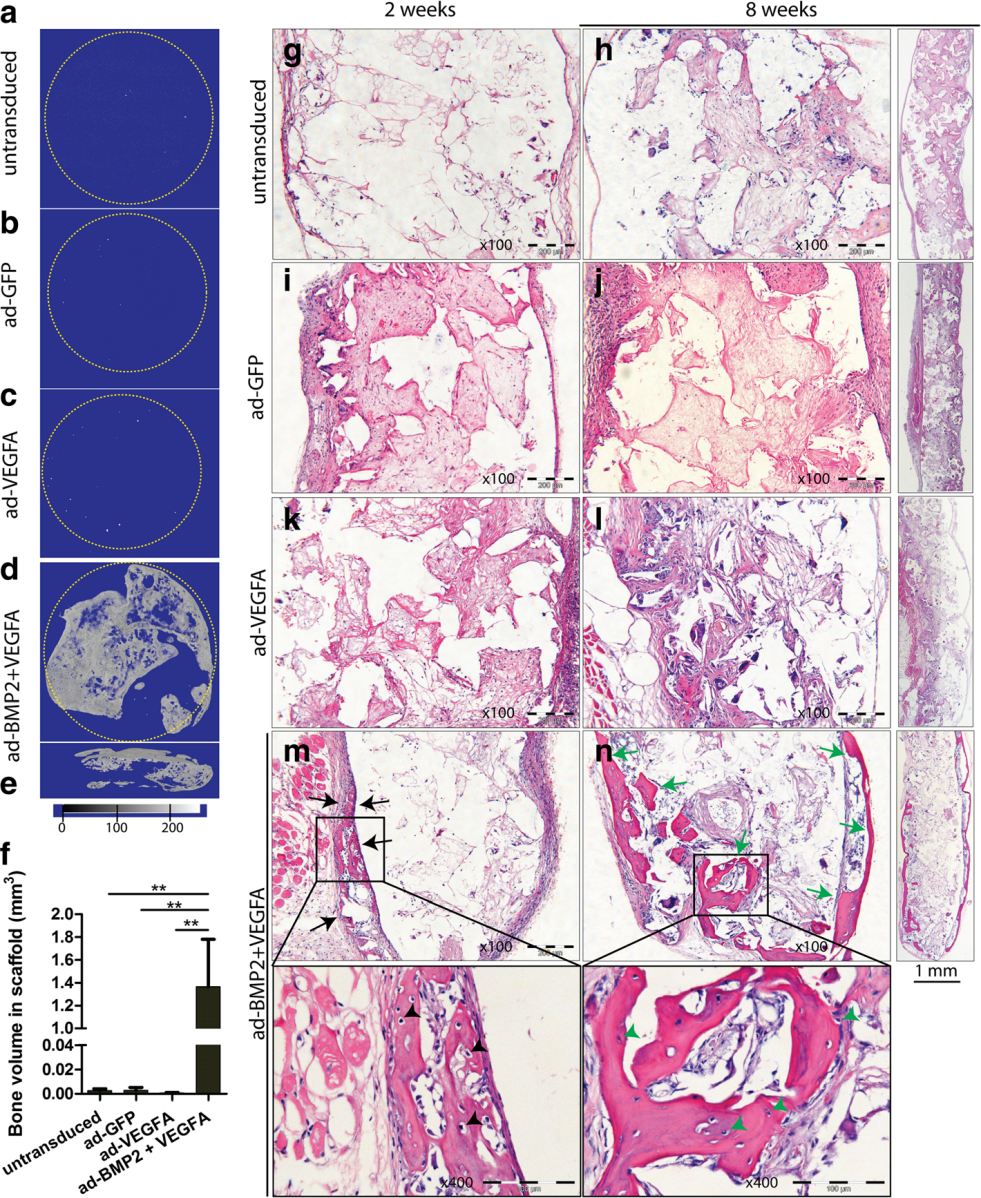
Combined delivery of bone morphogenetic protein 2 (BMP2) and vascular endothelial growth factor A (VEGFA) induced ectopic bone formation in scaffold explants in a subcutaneous NOD/SCID mouse model. The ability of VEGFA alone or in combination with BMP2 to induce ectopic bone formation was investigated in NOD/SCID mice by subcutaneously implanting scaffolds seeded with 5 × 10^5^ BMSC from different experimental groups. Scaffold explants at 8 weeks were examined by μCT to evaluate ectopic bone formation. Negligible amounts of radiopaque bone-like structures were detected in **a** untransduced, **b** ad-GFP, and **c** ad-VEGFA BMSC. In contrast, numerous bony-like radiopaque structures were found at the periphery as well as within the scaffolds of ad-BMP2 + VEGFA explants (**d**, cross sectional view in **e**). **f** Quantification of the radiopaque bone-like structures in the scaffold explants revealed significant amounts of bone formation in ad-BMP2 + VEGFA scaffolds. **g**–**n** Representative images of H&E stained FFPE sections of scaffold explants from different experimental groups at 2 and 8 weeks. **g**–**l** Formation of bony structures was not detected in untransduced, ad-GFP, or ad-VEGFA groups at either 2 or 8 weeks. **m** In the ad-BMP2 + VEGFA BMSC group, formation of bony structures (black arrows) could be seen as early as 2 weeks, mostly at the periphery of the scaffold explants. **n** Extensive bone formation (green arrows) with bony trabeculae extending inside the scaffolds was found at 8 weeks in ad-BMP2 + VEGFA BMSC. The structure consisted of numerous osteocyte-like cells at both 2 (black arrowheads, inset in **m**) and 8 weeks (green arrowheads, inset in **n**). ANOVA with Bonferroni post hoc analysis was carried out for statistical analysis in (**f**). Error bars represent SEM. **p < 0.01. ad adenoviral, GFP green fluorescent protein

The ability of VEGFA alone or in combination with BMP2 to induce ectopic bone formation in scaffolds was investigated by subcutaneously implanting unseeded scaffolds (scaffold-only), or scaffolds seeded with untransduced or with ad-GFP, ad-VEGFA, and ad-BMP2 + VEGFA BMSC. μCT analysis revealed barely detectable radiopaque, bone-like structures in the scaffold-only explants (data not shown), untransduced (0.0026 ± 0.0014 mm^3^), ad-GFP (0.0029 ± 0.0023 mm^3^), or ad-VEGFA (0.0008 ± 0.0002 mm^3^) groups at 8 weeks (Fig. 4a–c, f). In contrast, all analyzed replicates from ad-BMP2 + VEGFA explants at 8 weeks revealed the formation of dense bone-like structure (1.37 ± 0.414 mm^3^) on the periphery as well as within the scaffold explant (Fig. 4d–f). Histological examination of H&E stained sections was next performed to confirm the formation of bone structures in ad-BMP2 + VEGFA explants. As with the μCT findings, no bony structures were detected in any replicates of untransduced, ad-GFP, or ad-VEGFA explants at either 2 or 8 weeks (Fig. 4g–l). However, formation of bony structures was detected at the periphery of the scaffold explants in all replicates (7/7) of ad-BMP2 + VEGFA BMSC at 2 weeks (Fig. 4m). At 8 weeks, formation of bony structures was more extensive, with bony trabeculae in all the replicates examined (6/6) (Fig. 4n). The bony structure consisted of numerous osteocyte-like cells at both 2 and 8 weeks. Occasional inflammatory and multinucleated giant cells were detected in all groups (Fig. 4g–n).

### Combined delivery of VEGFA- and BMP2-mediated bone formation is associated with upregulation of osteogenic and angiogenic molecules in vivo

**Fig. 5:**
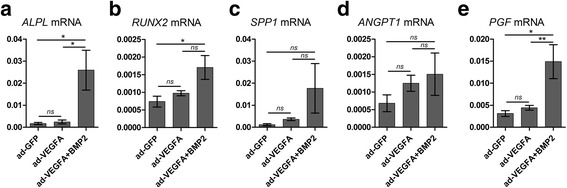
Bone morphogenetic protein 2 (BMP2) and vascular endothelial growth factor A (VEGFA) co-expression-mediated bone formation is associated with upregulation of osteogenic and angiogenic markers in the in vivo scaffold explants. Custom PCR array was used to examine differentially expressed osteogenesis- and angiogenesis-related genes in scaffold explants at 2 weeks. ANOVA revealed upregulation of a number of osteogenesis- and angiogenesis-related genes such as **a** *ALPL*, **b** *RUNX2*, **c** *SPP1* (*osteopontin*), **d** *ANGPT1*, and **e** *PGF* in ad-BMP2 + VEGFA explants compared with the ad-GFP and ad-VEGFA explants. ANOVA test with Bonferroni post hoc analysis was undertaken for statistical analysis. **p < 0.01, **p < 0.05. GFP green fluorescent protein, ns not significant

Custom PCR array was used to examine differentially expressed osteogenesis- and angiogenesis-related genes in scaffold explants at 2 weeks. ANOVA showed upregulation of osteogenesis-related genes such as *ALPL*, *RUNX2*, and *SPP1* in ad-BMP2 + VEGFA explants compared with the ad-GFP and ad-VEGFA explants (Fig. 5a–c). Moreover, *ANGPT1*, an angiogenic factor which modulates endothelial differentiation, and *PGF* were found to be upregulated in ad-BMP2 + VEGFA explants (Fig. 5d, e). mRNA levels of *RUNX2*, *SPP1*, *ANGPT1*, and *PGF* were moderately but nonsignificantly induced in the ad-VEGFA explants compared with the ad-GFP explants (Fig. 5b–e).

### Comparison of mono-delivery of BMP2 and VEGFA and combined delivery of BMP2 and VEGFA in angiogenesis and osteogenesis in vivo

The animal experiments in both the current investigation and our previous study (using adenoviral-mediated mono-delivery of BMP2 (ad-BMP2) [13] were carried out at the same time by the same authors (SS, YX, and DS) and using the same methodology. This allows comparison of the angiogenic and osteogenic effects of mono-delivery of BMP2 [13] with those of the experimental groups in the current study. The percentage of CD31-positive area (using anti-mouse CD31) was found to be lower (14.31 ± 2.07%) in ad-BMP2 compared with ad-VEGFA (40.17 ± 3.17%) and ad-BMP2 + VEGFA (25.89 ± 2.5%) explants at 2 weeks. Nevertheless, the amount of bone-like radiopaque structures was found to be significantly higher in ad-BMP2 (7.37 ± 0.5834 mm^3^) than in ad-VEGFA (0.0008 ± 0.0002 mm^3^) and ad-BMP2 + VEGFA (1.37 ± 0.414 mm^3^) explants at 8 weeks.

## Discussion

We previously established a model using adenoviral-mediated delivery of BMP2 to human BMSC seeded onto recently developed and characterized poly(LLA-co-CL) scaffolds. We showed that adenoviral-mediated expression of BMP2 significantly enhanced in vitro osteogenic differentiation and in vivo bone forming ability of BMSC [13]. Moreover, BMP2 induced pre-angiogenic transcriptional programs in BMSC, both in vitro and in vivo [13]. In the present study, using the above model, we demonstrated that BMSC expressing VEGFA alone and in combination with BMP2 significantly induced angiogenesis. However, in contrast to the combined delivery, VEGFA alone failed to demonstrate osteogenic activity both in vitro and in vivo in a subcutaneous mouse model.

Although, a wide range of VEGFA/BMP2 ratios have been tested for their effectiveness in bone regeneration, it has been proposed that delivery of a lower ratio of VEGF to BMP2 results in more efficient angiogenic and osteogenic activity in bone regeneration ([14] and references therein, [16]). Accordingly, the current study utilized a ratio of 1:3 of ad-VEGFA to ad-BMP2 to successfully express the respective mRNA and proteins in BMSC (Fig. 1f–i). Transcriptomic analysis using PCR array, independent validation using TaqMan-based qRT-PCR, and ALP assay showed upregulation of osteogenic markers *ALPL*, *RUNX2*, *BMP7*, *BMP6*, *BGLAP*, and *SPP1* at day 3 or 14 in vitro only in the ad-BMP2 + VEGFA BMSC compared with the ad-GFP and ad-VEGFA BMSC (Fig. 2c, d and Additional file 2: Figure S1A–D). These results suggest that although combined delivery of BMP2 and VEGFA was able to differentiate the BMSC grown in scaffolds towards an osteogenic pathway, VEGFA alone was not able to do so. Similar gene expression results for osteogenic markers were also found only in the ad-BMP2 + VEGFA BMSC from donors 2 and 3 (Additional file 2: Figure S1E–R), implying that the osteogenic effects of combined delivery of BMP2 and VEGFA were not restricted to a certain type of BMSC strain. Additionally, mRNA expression levels of angiogenesis-related molecules, such as *FLT1* (VEGFR1) and its ligand *PGF* were significantly upregulated in ad-BMP2 + VEGFA BMSC compared with the ad-GFP and ad-VEGFA BMSC (Fig. 3c, d). One possible explanation for the inability of the ad-VEGFA BMSC group to induce the expression of *FLT1* and *PGF* could be that, although they are angiogenesis-related molecules, the *FLT1* receptor and its selective ligand *PGF* are not the direct downstream targets of VEGFR2 activation [29]. Moreover, increased expression of *FLT1* and *PGF* only in ad-BMP2 + VEGFA BMSC as observed in the current study is very likely due to BMP2 expression, as shown in our previous study where adenoviral-mediated expression of BMP2 in BMSC led to higher expression of *FLT1* and *PGF* mRNA levels [13].

The angiogenic and osteogenic properties of adenoviral-mediated expression of VEGFA alone and in combination with BMP2 expression were subsequently investigated by subcutaneous implantation of scaffolds seeded with BMSC into NOD/SCID mice. On gross visual examination, scaffold explants from ad-VEGFA and ad-BMP2 + VEGFA BMSC groups at 2 weeks were more reddish in color, with many capillary/vessel-like structures radiating out from the scaffold explants compared with the explants from the control groups (Fig. 3a), indicating a richer vascular supply in these scaffold explants. Correspondingly, significantly higher numbers of CD31-positive (against mouse CD31) capillary-like structures were found in ad-VEGFA and ad-BMP2 + VEGFA explants than in the ad-GFP explants (Fig. 3b–d). Interestingly, the percentage of CD31-positive areas was found to be higher in ad-VEGFA than in the ad-BMP2 + VEGFA (in the current study) or ad-BMP2 explants [13]. The lower level of angiogenesis in ad-BMP2 + VEGFA explants is apparently in contrast to the fact that both VEGFA and BMP2 could induce angiogenesis. However, the influence of fewer ad-VEGFA virus particles used in ad-BMP2 + VEGFA (25 MOI, compared with 100 MOI used in ad-VEGFA group) could not be completely ruled out. It is noteworthy that very few capillary-like structures were weakly positive only in ad-VEGFA BMSC scaffold explants at 8 weeks when incubated with anti-human CD31 antibody (Additional file 3: Figure S2), suggesting that the induction of capillary formation was mediated largely by the paracrine effect of VEGFA, leading to the recruitment of mouse endothelial cells and/or differentiation of mouse progenitor cells to endothelial lineage in the scaffold explants, rather than by endothelial differentiation of the implanted BMSC (cell-autonomous effect). Accordingly, expression of human *PECAM1* was slightly, but insignificantly overexpressed in ad-VEGFA BMSC explants at 2 weeks (data not shown). Similar observations were made by Behr et al. using VEGFA-treated human adipose-derived stem cells [30]. Taken together, these findings indicate that both VEGFA alone or in combination with BMP2 enhances angiogenesis in scaffolds seeded with BMSC, primarily by recruitment and differentiation of mouse progenitor cells to the endothelial lineage and, secondarily, possibly by endothelial differentiation of the implanted BMSC.

After observing increased angiogenesis in ad-VEGFA and ad-BMP2 + VEGFA explants, the next step was to explore the osteogenic properties of VEGFA alone and in combination with BMP2. μCT analysis disclosed barely detectable radiopaque, bone-like structures in all the control groups and in the ad-VEGFA explants, whereas significant amounts were detected in the ad-BMP2 + VEGFA explants (Fig. 4a–f). In accordance with these results, histological examination of H&E stained sections showed no bony structures in the scaffold explants from any of the control groups, including ad-VEGFA BMSC, at either 2 or 8 weeks (Fig. 4g–l). In contrast, formation of bony structures was observed in ad-BMP2 + VEGFA scaffold explants as early as 2 weeks, with further bone formation observed at 8 weeks (Fig. 4m, n). The presence of numerous osteocyte-like cells in the bony structures in ad-BMP2 + VEGFA explants (Fig. 4m, n, insets) suggests that the newly formed bone in the scaffolds was indeed vital tissue. In corroboration, mRNA expression levels of *ALPL*, *RUNX2*, and *SPP1* were significantly upregulated only in ad-BMP2 + VEGFA explants. That ad-VEGFA BMSC failed to form bone in the scaffold explants is in accordance with some previous studies in which expression of VEGFA either had no osteogenic effect alone, or had no additional osteogenic effects over BMP2 alone [16, 21, 31]. However, these observations are inconsistent with some studies which have shown osteogenic properties of VEGFA [9, 32]. These differences could be attributable to various factors, such as the delivery system (controlled release versus gene therapy), the VEGFA dose, the in vivo model system used, assessment time points, and the local microenvironment. Additionally, it is worth mentioning that ad-BMP2 exhibited the highest degree of bone formation, even though it was associated with the moderate induction of angiogenesis (least degree of angiogenesis compared with ad-VEGFA and ad-BMP2 + VEGFA explants). This indicates that an optimal degree of angiogenesis might be necessary in bone regeneration. In line with this suggestion, previous studies have reported that VEGFA-mediated excessive angiogenesis was associated with deleterious effects in muscles [33, 34] and embryonic myocardium development [35].

## Conclusions

Using the previously established model system [13], the results of this study demonstrate that BMSC expressing VEGFA alone and in combination with BMP2 significantly induced angiogenesis. However, compared with the combined delivery, VEGFA alone failed to demonstrate osteogenic activity both in vitro and in vivo in a subcutaneous mouse model. These findings not only call into question the use of VEGFA alone for bone regeneration, but also highlight the importance of appropriately formulated combined delivery of VEGFA and BMP2.

## Additional files

Additional file 1: Supplementary materials, methods, and results. (DOCX 13 kb)

Additional file 2: Figure S1. Combined delivery of BMP2 and VEGFA induced upregulation of ALPL and RUNX2 mRNA levels in ad-BMP2 + VEGFA BMSC from donors 2 and 3. Independent validation of the differentially expressed selected genes *(ALPL*, *RUNX2*, or *SPP1)*, as identified by PCR array, was achieved by performing TaqMan-based qRT-PCR for BMSC from all donors. Compared with the controls, mRNA levels of *BMP2*, *VEGFA*, *ALPL*, *RUNX2*, or *SPP1* were significantly overexpressed at days 3 or 14 in ad-BMP2 + VEGFA BMSC from donor 1 (A–D), donor 2 (E–K), and donor 3 (L–R) seeded in scaffolds. Error bars represent SEM of three biological replicates (*n* = 3) performed in three technical replicates. ANOVA with Bonferroni post hoc analysis was performed for statistical analysis. ***p < 0.001; **p < 0.01; *P < 0.05. ns not significant. (TIF 2367 kb)

Additional file 3: Figure S2. A limited number of blood capillaries, only in ad-VEGFA scaffold explants, were weakly positive for anti-CD31 antibody targeting human CD31 protein. (B–G) No CD31-positive staining was observed in the capillary/vessel-like structures (black arrows) in the entire scaffold explants from all groups both at 2 and 8 weeks, except for a few capillaries in the ad-VEGFA explants at 8 weeks (E, green arrows). (A) Positive control (normal human oral mucosa) showed multiple CD31-positive capillary-like structures. (TIF 14626 kb)

## Abbreviations

ALP: Alkaline phosphatase
ANOVA: Analysis of variance
BMP: Bone morphogenetic protein
BMSC: Bone marrow stromal cells
BSA: Bovine serum albumin
BTE: Bone tissue engineering
eGFP: Enhanced green fluorescent protein
ELISA: Enzyme-linked immunosorbent assay
FFPE: Formalin fixed and paraffin embedded
H&E: Hematoxylin and eosin
IF: Immunofluorescence
IHC: Immunohistochemistry
μCT: Microcomputed tomography
MOI: Multiplicity of infection
NOD/SCID: Nonobese diabetic/severe combined immunodeficiency
PBS: Phosphate-buffered saline
poly(LLA-co-CL): Poly (L-lactide-co-є-caprolactone)
qRT-PCR: Quantitative reverse-transcription polymerase chain reaction
VEGFA: Vascular endothelial growth factor A
