# Supplementary material for: Delivery of VEGFA in bone marrow stromal cells seeded in copolymer scaffold enhances angiogenesis, but is inadequate for osteogenesis as compared with the dual delivery of VEGFA and BMP2 in a subcutaneous mouse model
Source: Stem Cell Res Ther. 2018 Jan 31;9:23. doi: 10.1186/s13287-018-0778-4 (PMC5793460; doi:10.1186/s13287-018-0778-4)
Supplement: Supplementary file 1 — Supplementary materials, methods, and results. (DOCX 13 kb) [file 13287_2018_778_MOESM1_ESM.docx]

Additional file 1

Material and Methods

Cell Culture

For validation of the gene expression changes induced by adenoviral mediated delivery of VEGFA alone and in combination with BMP2 in the donor 1 BMSC, human BMSC from two additional sources were used (henceforth referred to as donors 2 and 3 BMSC). Donor 2 BMSC were isolated, characterized and expanded from human bone marrow aspirates purchased from Lonza Walkersville Inc., MD, USA. The cells were characterized by flow cytometry, which showed that more than 90% of cells expressed stem cell surface markers of CD105, CD90, CD73 and fewer than 4% expressed CD34 and CD45 surface markers. Donor 3 BMSC were purchased from Lonza (Cat. number: PT-2501, Lonza Walkersville Inc., MD, USA). BMSC from donor 2 and 3 were grown in MSCGM^TM^ Mesenchymal stem cell growth medium (Cat. number: PT-3001, Lonza Walkersville Inc., MD, USA).

**Results**

*Validation of combined delivery of BMP2 and VEGFA mediated up-regulation of ALPL and RUNX2 mRNA expression levels in ad-BMP2 + VEGFA BMSC from donors 2 and 3*

To confirm whether the combined delivery of BMP2 and VEGFA induces expression of similar osteogenic genes in BMSC from other donors, BMSC from donors 2 and 3 were infected with respective adenoviral particles and examined for mRNA expression levels of *ALPL* and *RUNX2* by TaqMan qRT-PCR. BMSC grown in rhVEGFA physiosorbed scaffolds (100 ng/scaffold) served as additional controls. Similar to donor 1 BMSC, mRNA levels of *BMP2*, *VEGFA,* *ALPL* or *RUNX2* were over-expressed at day 3 or 14 in ad-BMP2 BMSC from donors 2 and 3 grown in 3D scaffolds, compared with the control ad-GFP BMSC (Fig. S1E-R).

Fig. S1

*Combined delivery of BMP2 and VEGFA induced up-regulation of ALPL and RUNX2 mRNA levels in ad-BMP2 + VEGFA BMSC from donors 2 and 3*

Independent validation of the differentially expressed selected genes *(ALPL, RUNX2* or *SPP1)*, as identified by PCR array, was done by performing TaqMan based qRT-PCR for BMSC from all donors. Compared with the controls, mRNA levels of *BMP2*, *VEGFA*, *ALPL*, *RUNX2* or *SPP1* were significantly over-expressed at day 3 or 14 in ad-BMP2 + VEGFA BMSC from donor 1 (A-D), donor 2 (E-K) and donor 3 (L-R) seeded in scaffolds. Error bars represent SEM of 3 biological replicates (*n*=3) done in 3 technical replicates. ANOVA with Bonferroni *post hoc* analysis was performed for statistical analysis. ***, *p*<0.001; **, *p=*0.001-0.01; *, *P*=0.01-0.05; ns, non-significant.

Fig. S2

*A limited number of* *blood capillaries, only in ad-VEGFA scaffold explants, were weakly positive for anti-CD31 antibody targeting human CD31 protein*

(B-G) No CD31 positive staining was observed in the capillary/vessels like structures (black arrows) in the entire scaffold explants from all groups both at 2 and 8 weeks, except for a few capillaries in the ad-VEGFA explants at 8 weeks (E, green arrows). (A) Positive control (normal human oral mucosa) showed multiple CD31 positive capillary like structures.
