# Supplementary figures and images for: Delivery of VEGFA in bone marrow stromal cells seeded in copolymer scaffold enhances angiogenesis, but is inadequate for osteogenesis as compared with the dual delivery of VEGFA and BMP2 in a subcutaneous mouse model
Source: Stem Cell Res Ther. 2018 Jan 31;9:23. doi: 10.1186/s13287-018-0778-4 (PMC5793460; doi:10.1186/s13287-018-0778-4)

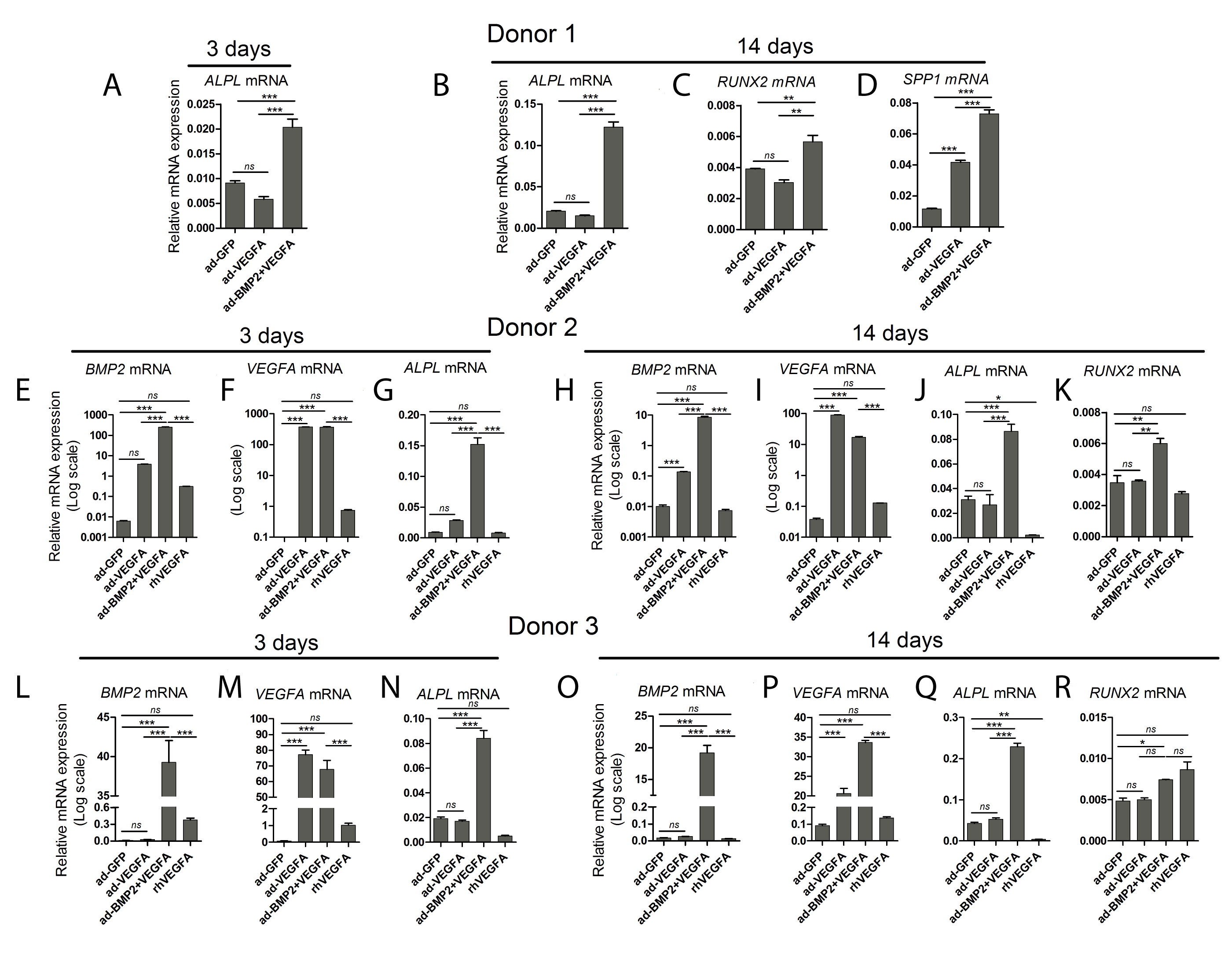

Supplement: Supplementary file 2 — Combined delivery of BMP2 and VEGFA induced upregulation of ALPL and RUNX2 mRNA levels in ad-BMP2 + VEGFA BMSC from donors 2 and 3. Independent validation of the differentially expressed selected genes (ALPL, RUNX2, or SPP1), as identified by PCR array, was achieved by performing TaqMan-based qRT-PCR for BMSC from all donors. Compared with the controls, mRNA levels of BMP2, VEGFA, ALPL, RUNX2, or SPP1 were significantly overexpressed at days 3 or 14 in ad-BMP2 + VEGFA BMSC from donor 1 (A–D), donor 2 (E–K), and donor 3 (L–R) seeded in scaffolds. Error bars represent SEM of three biological replicates (n = 3) performed in three technical replicates. ANOVA with Bonferroni post hoc analysis was performed for statistical analysis. ***p < 0.001; **p < 0.01; *P < 0.05. ns not significant. (TIF 2367 kb) [file 13287_2018_778_MOESM2_ESM.tif]

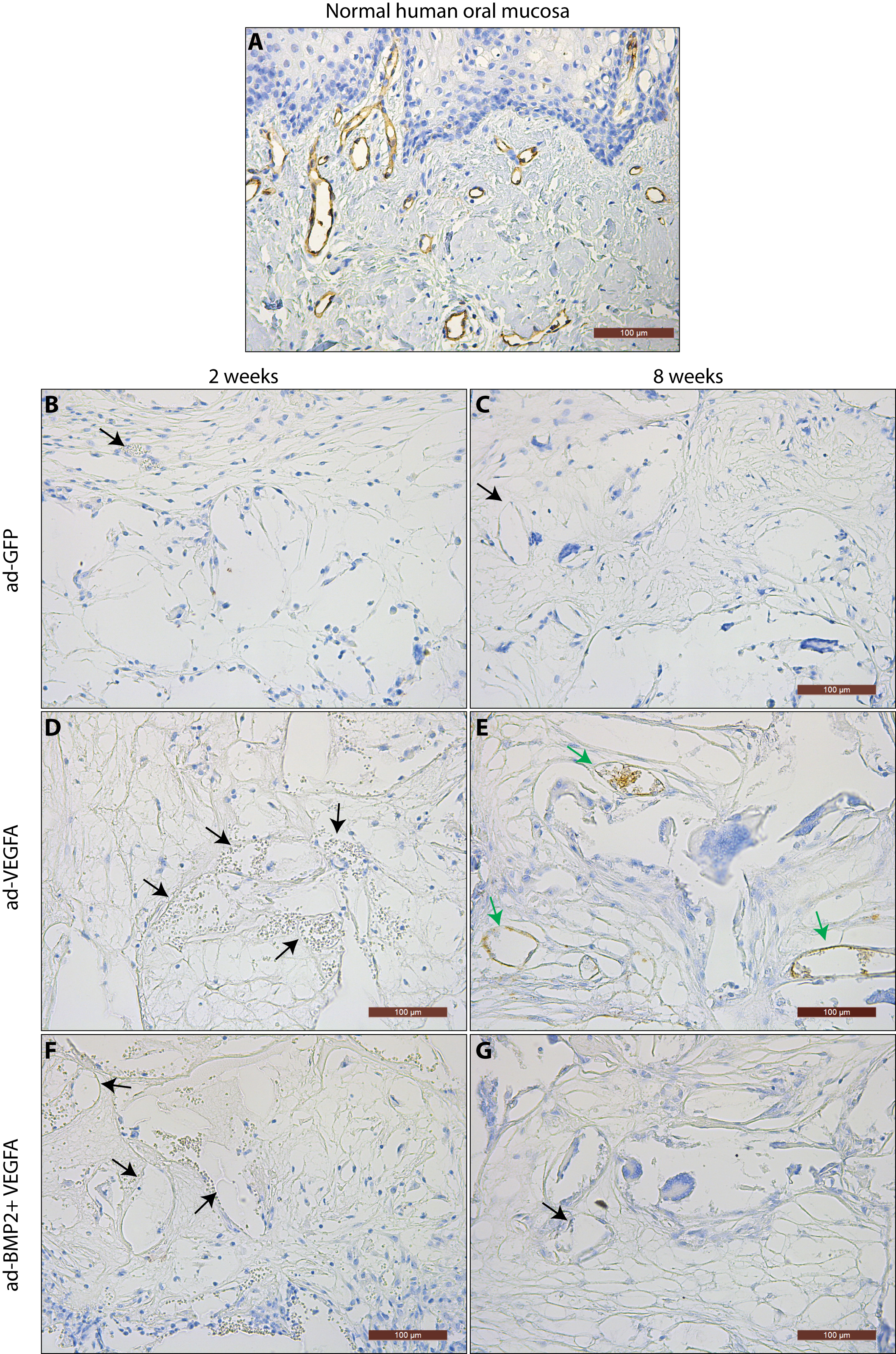

Supplement: Supplementary file 3 — A limited number of blood capillaries, only in ad-VEGFA scaffold explants, were weakly positive for anti-CD31 antibody targeting human CD31 protein. (B–G) No CD31-positive staining was observed in the capillary/vessel-like structures (black arrows) in the entire scaffold explants from all groups both at 2 and 8 weeks, except for a few capillaries in the ad-VEGFA explants at 8 weeks (E, green arrows). (A) Positive control (normal human oral mucosa) showed multiple CD31-positive capillary-like structures. (TIF 14626 kb) [file 13287_2018_778_MOESM3_ESM.tif]
